# Isolated Granulocytic Sarcoma of the Breast after Allogeneic Stem Cell Transplantation: A Rare Involvement Also Detected by 18FDG-PET/CT

**DOI:** 10.4274/Tjh.2012.0106

**Published:** 2014-03-05

**Authors:** Eren Gündüz, Meltem Olga Akay, Mustafa Karagülle, İlknur Sivrikoz Ak

**Affiliations:** 1 Eskişehir Osmangazi University School of Medicine, Department of Hematology, Eskişehir, Turkey; 2 Eskişehir Osmangazi University School of Medicine, Nuclear Medicine, Eskişehir, Turkey

**Keywords:** Granulocytic sarcoma, Breast, stem cell transplantation, 18FDG-PET/CT

## Abstract

Granulocytic sarcoma is a tumor consisting of myeloid blasts with or without maturation that occurs at an anatomical site other than bone marrow. Most frequently affected sites are skin, lymph nodes, gastrointestinal tract, bone, soft tissue and testes. AML may manifest as granulocytic sarcoma at diagnosis or relapse. Although it has been considered to be rare relapse as granulocytic sarcoma after stem cell transplantation is being increasingly reported. However it is rare without bone marrow involvement and in AML M6 subtype. Breast is also a rare involvement. We report a 30-year-old woman with AML M6 relapsed 16 months after allogeneic stem cell transplantation as a granulocytic sarcoma in right breast without bone marrow involvement. She was treated with systemic chemotherapy but died of sepsis. 18FDG-PET/CT images were also obtained and detected lesions other than detected by breast ultrasound. The incidence of granulocytic sarcoma may increase if suspected or new diagnostic modalities are performed.

## Abstract (tr)

Granülositik sarkom matürasyonlu ve matürasyonsuz miyeloid blastlardan oluşan ve kemik iliği dışındaki anatomik bölgelerde yerleşen bir tümördür. En sık etkilenen bölgeler cilt, lenf nodları, gastrointestinal sistem, kemik, yumuşak doku ve testistir. AML tanı ya da relaps anında granülositik sarkom olarak ortaya çıkabilir. Nadir olduğu düşünülmekle birlikte kök hücre nakli sonrası granülositik sarkom olarak relaps giderek artan biçimde bildirilmektedir. Fakat kemik iliği tutulumu olmaksızın ve AML M6 alt tipinde nadirdir. Yazımızda AML M6 tanısıyla takip edilen ve allogeneik kök hücre naklinden 16 ay sonra kemik iliği tutulumu olmaksızın sağ memede granülositik sarkom şeklinde relaps görülen 30 yaşındaki kadın hastayı sunduk. Hastaya sistemik kemoterapi verildi ancak sepsis nedeniyle kaybedildi. 18FDG-PET/CT görüntülerinde meme ultrasonunda saptanmayan lezyonlar izlendi. Şüphe edildiği takdirde ya da yeni tanı modaliteleri kullanıldığında granülositik sarkom insidansının artabileceği kanaatindeyiz.

## INTRODUCTION

Allogeneic hematopoietic stem cell transplantation (allo SCT) decreases relapse risk and improves survival in unfavorable-risk acute myeloid leukemia (AML) patients [1]. Some patients with advanced AML can also achieve long-term survival [2]. Transplant-related mortality has decreased, but relapse after transplantation has emerged as the principle cause of treatment failure [3]. Extramedullary (EM) relapse of AML occurs in 5% to 7% of allo SCT recipients and accounts for 7% to 46% of total relapses [4]. AML M6 represents less than 5% of AML cases and its EM presentation is extremely rare [5,6,7]. We report a case of AML French-American-British (FAB) classification type M6 with relapse 16 months after allo SCT as a granulocytic sarcoma in the right breast without bone marrow involvement. 18Fluoro-deoxy-glucose positron emission tomography (18FDG-PET)/computed tomography (CT) images were also obtained as a tool for detection of EM relapse of AML. Informed consent was obtained.

## CASE REPORT

In December 2009, a 30-year-old woman was referred to our hospital because of pancytopenia, and a diagnosis of AML M6 type was made. At the time of diagnosis hemoglobin was 93 g/L, white blood cell count was 1.5x109/L, and platelet count was 60x10^9^/L. Biochemical tests other than lactate dehydrogenase (LDH) level were normal (LDH: 485 U/L, range: 240-480). Blasts in the bone marrow aspirate were negative for CD56. Cytogenetic analysis showed normal karyotype. EM leukemia was not demonstrated. She was treated with idarubicin at 12 mg/m2/day intravenously (iv) on days 1-3 and cytarabine (ara-C) at 100 mg/m2/day iv on days 1-7. Since complete remission (CR) was not detected, a second course of the same therapy was given. After achieving CR, consolidation therapy with ara-C at 3 g/m2/day iv on days 1.3 and 5 was administered. A bone marrow aspiration was performed in August 2010 because of thrombocytopenia. The result was compatible with AML relapse and she received ara-C at 6 g/m2/day iv on days 1, 3, 5, and 7; etoposide at 75 mg/m2/day iv on days 1-7; and idarubicin at 12 mg/m2/day iv on days 1-3.

In November 2010 the patient underwent an allo SCT from her human leukocyte antigen (HLA)-matched brother after a conditioning regimen of busulfan (16 mg/kg) and cyclophosphamide (120 mg/kg). Graft-versus-host disease (GVHD) prophylaxis consisted of cyclosporine and cyclophosphamide at 50 mg/kg/day on days 3 and 4. Full donor chimerism was obtained on day 28. Acute hepatic GVHD disappeared with methyl prednisolone therapy. Chronic GVHD confined to skin was treated with mycophenolate mofetil.

**Figure 1:**
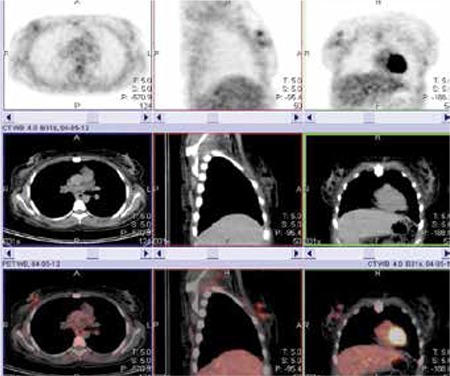
The patient was scanned by an integrated PET/CT camera (one hour after the administration of 465 MBq FDG), which consists of a 6-slice CT gantry integrated on a LSO based full ring PET scanner (Siemens Biograph 6, IL, Chicago, USA). MIP PET, CT and fusion PET/CT images were obtained. There were 2 focal lesions with moderate metabolic activity (SUV max of 3.6) in the upper inner quadrant of the right breast.

In April 2012 she was admitted with a palpable mass in the right breast. The breast ultrasound showed an approximately 33-mm irregular mass with heterogeneous internal echo suggesting carcinoma of the breast. She underwent an excisional biopsy and the diagnosis was granulocytic sarcoma. Bone marrow aspiration and biopsy revealed no involvement. Chimerism was still of the full donor type. ^18^FDG-PET/CT was performed after biopsy. The time between ^18^FDG-PET/CT and the biopsy was 32 days. There were 2 focal lesions with moderate metabolic activity (standardized uptake value maximum [SUV max] of 3.6) in the upper inner quadrant of the right breast (Figure 1). CT images alone were not definitive. Since the time between ^18^FDG-PET/CT and the biopsy was 32 days and the margin of the hyperactive lesions were regular, the nuclear medicine physician concluded that the lesions were not related with postoperative changes but that they were true masses.

In May 2012 she was given high-dose ara-C, etoposide, and idarubicin combination chemotherapy again. Invasive aspergillosis developed despite posaconazole prophylaxis and she died of sepsis 25 days after chemotherapy.

## DISCUSSION

Recent studies have suggested that EM relapse accounts for a significant proportion of relapses after allo SCT and is particularly associated with the induction of graft-versus-leukemia (GVL) effect [4]. Younger age, EM involvement before SCT, advanced disease at SCT, unfavorable cytogenetics, and M4 and M5 FAB subtypes are factors reported to be associated with EM relapse after SCT [8,9]. The significance of chromosomal abnormalities such as t(8;21) and inv (16), CD56 expression in leukemic cells, T-cell depletion of grafts, stem cell sources, HLA disparities, kinetics of T-cell chimerism, and the preventive role of total body irradiation remains unclear [4]. None of the reported factors except younger age were recognized in our patient, and so further studies are needed to clarify the role of other factors in EM relapse. 

The median time from SCT to EM relapse has been reported as 10 to 17 months [8,10,11]. This time was 16 months in our patient. Lee et al. [12] indicated that the GVL effect associated with an occurrence of GVHD is less effective in preventing an EM relapse than a bone marrow relapse. This seems also true for our patient because acute hepatic and chronic skin GVHD granulocytic sarcoma occurred without bone marrow involvement. Unfortunately, we were unable to evaluate the occurrence of bone marrow relapse because the patient died early.

Since the diagnosis of EM relapse is often delayed, new diagnostic modalities such as ^18^FDG-PET/CT are discussed with several limitations. This method can identify new extramedullary manifestations that were not clinically detectable, but it should be applied not just during diagnosis but also for the assessment of treatment response and for detecting recurrence [13]. Reported ^18^FDG uptake ranges between SUVmax 2.6 and 9.7 [14]. The SUVmax was 3.6 in our patient. Although the breast ultrasound showed just one mass and ^18^FDG-PET/CT was performed after excisional biopsy, two different lesions were detected by ^18^FDG-PET/CT. This seems to be an advantage because masses that cannot be demonstrated with conventional methods can be seen in ^18^FDG-PET/CT, and this is an important issue, especially for isolated EM relapses. Unfortunately, ultrasound is known to be a user-dependant method and we did not use any more specific methods like magnetic resonance imaging. 

Although local therapy, including surgical excision and radiotherapy, can offer long-term survival for some patients, most patients develop systemic relapse. Thus, systemic or combined modality therapy should be considered, particularly in patients with good performance status [10]. Donor lymphocyte infusion (DLI) has limited efficacy [14] and a second transplant often results in repeated relapse [15]. Our patient’s performance was good enough for combined modality treatment and we planned systemic chemotherapy followed by DLI. The combination was chosen as the one that we managed best before transplantation. However, the patient died of sepsis after chemotherapy although she tolerated the same combination well before transplantation. This may be because the combination has become more toxic after the effects of allo SCT and its complications.

In conclusion, the prognosis of patients who develop EM relapse after allo SCT remains poor. The number of EM relapses will be increasing as the number of transplant patients increases. Transplant physicians should be aware of EM relapses and the diagnosis should be made as early as possible. New diagnostic modalities such as ^18^FDG-PET/CT for early diagnosis and new agents may improve clinical outcome. 

## CONFLICT OF INTEREST STATEMENT

The authors of this paper have no conflicts of interest, including specific financial interests, relationships, and/ or affiliations relevant to the subject matter or materials included.
